# Long-term neuromuscular, cardiac and liver outcomes in an adult man affected with Chanarin-Dorfman syndrome

**DOI:** 10.1016/j.ymgmr.2025.101265

**Published:** 2025-10-07

**Authors:** Kinza Noman, Andreas Tridimas, James B. Lilleker, Gaetano Nucifora, Peter Woolfson, Daniel du Plessis, Alison Woodall, Andrew Oldham, Mark E. Roberts, John Bassett, Federico Roncaroli, Simon A. Jones, Stefan Coassin, Florian Kronenberg, Karolina M. Stepien

**Affiliations:** a Diabetes and Endocrinology Department, St Mary's Hospital, Praed St, London W2 1NY, UK; b Clinical Biochemistry Department, Countess of Chester Hospital, Chester, UK; c Manchester Centre for Clinical Neuroscience, Northern Care Alliance NHS Foundation Trust, Manchester Academic Health Sciences Centre, Salford, UK; d Centre for Musculoskeletal Research, Division of Musculoskeletal and Dermatological Sciences, School of Biological Sciences, The University of Manchester Faculty of Biology Medicine and Health, Manchester, UK; e Cardiac MRI Unit, North West Heart Centre, Wythenshawe Hospital, Manchester Foundation NHS Foundation Trusts, Manchester, UK; f Division of Cardiovascular Sciences, University of Manchester, Manchester M13 9PL, UK; g Cardiology Department, Salford Royal Hospital, Northern Care Alliance NHS Foundation Trust, Manchester, UK; h Department of Cellular Pathology, Northern Care Alliance, Salford Royal NHS Foundation Trust, Salford, UK; i Adult Inherited Metabolic Diseases, Salford Royal Organisation, Northern Care Alliance NHS Foundation Trust, Stott Lane, M6 8HD, Salford, Greater Manchester, UK; j Geoffrey Jefferson Brain Research Centre, Division of Neuroscience, Faculty of Biology, Medicine and Health, University of Manchester, Manchester, UK; k Willink Unit, Manchester Centre for Genomic Medicine, St Mary's Hospital, Manchester Academic Health Sciences Centre, University of Manchester, CMFT, Manchester, UK; l Institute of Genetic Epidemiology, Medical University of Innsbruck, Innsbruck, Austria

**Keywords:** Chanarin–Dorfman syndrome, CGI-58, liver disease, Ichthyosis, neuromuscular disease, neutral lipid storage disease

## Abstract

Chanarin-Dorfman syndrome (CDS) is an ultra-rare autosomal recessive subtype of neutral lipid storage disorder (NLSD); it is characterised by ichthyosis and intracytoplasmic accumulation of lipid droplets containing triglycerides, most commonly in granulocytes, muscle fibres, skin and liver. Several pathogenic variants in the *ABHD5*/CGI-58 gene have been described. Clinical manifestations include steatohepatitis, myopathy, ophthalmic disease, developmental delay. Liver involvement is an important cause of morbidity and mortality.

We present a case of a 26-year-old male diagnosed with ichthyotic NLSD in childhood, who developed progressive myopathy and cardiac fibrosis in adulthood. He was treated with a combination of low-fat diet, MCT oil and co-enzyme Q10 which resulted in an initial improvement in muscle strength and stabilisation of muscle symptoms and well-being.

Synopsis: Medical and dietetic management of liver and muscle complications in Chanarin-Dorfman syndrome.

## Highlights

• Marked vacuolation of neutrophils on blood film is consistent with Jordan's anomaly
• Ichthyosis, raised creatine kinase, Jordan's anomaly and new onset myopathy are pathognomonic features of with Chanarin-Dorfman syndrome.
• Lipid accumulation in type 1 and 2 fibres may be a diagnostic feature of the condition
• The severity of the generalized ichthyosis is not correlated with the presence of lipid droplets in the epidermis
• A low-fat diet, ursodeoxycholic acid and vitamin E can be recommended for patients with liver involvement.
• Regular cardiac MRI is important for monitoring Chanarin-Dorfman syndrome.

## 1 Introduction

Chanarin-Dorfman syndrome (CDS) (OMIM #275630) is an ultra-rare autosomal recessive subtype of neutral lipid storage disorder (NLSD) presenting as congenital ichthyosis [1] Impaired lipolysis leads to a spectrum of progressive multi-systemic symptoms. Myopathy (60 %) [2] and steatohepatitis (60–70 %) are common [1,3]. Variable multi-organ involvement has been documented including splenomegaly (19–24 %) [1,4] sensorineural hearing loss, ataxia, eye disease (42 %; including nystagmus, strabismus, cataract, retinal dysfunction), microcephaly, mental retardation (8 %) [1], rickets, eccrine gland vacuolation and growth retardation [1,3] Patients are often born as collodion babies, occasionally accompanied by bilateral ectropion (29 %) [1], and eclabium [5].^.^

Several phenotypes of the condition have been described. Pathogenic variants in *ABHD5*/CGI-58 are associated with CDS while pathogenic variants in adipose triglyceride lipase (*ATGL*; known as PNPLA2) cause NSLD associated with skeletal myopathy and cardiomyopathy [6]. The main difference between CDS and non-ichthyotic NSLD is that the lack of CGI-58 results in ichthyosis, whereas absence of ATGL causes cardiac myopathy [7].

Lipid vacuoles in neutrophils known as Jordan's anomaly identified in blood films and marrow smears are diagnostic for the condition [8] along with pathogenic variants in the hydrolase (EC * 604780) domain containing (*ABHD5*) 5 genes on the 3p21 chromosome [1,3,9,10]. It has been also postulated that *ABHD5* may interact with perilipin, a protein that regulates the breakdown of triacylglycerol in lipid droplets [11].

The neurologic manifestations in non-ichthyotic NSLD, such as cognitive impairment and psychiatric disorders, provides evidence for an ATGL independent function of CGI-58, presumably in the homeostasis of brain lipids [6].

Of approximately 120 cases of CDS described to date [1,12,13]. Each of the cases has a unique pathogenic variant leading to phenotypic variability [[14], [15], [16]]. This case report outlines the outcome of an adult patient and the progression of his myopathy.

## 2 Case

A 26-year-old Southeast Asian male, born to consanguineous parents at 35 weeks, was diagnosed with NLSD in childhood. He presented with an ichthyotic eczematous rash on his face and received intensive treatment for acute respiratory chest infections and gastrointestinal symptoms *e.g.* diarrhoea and abdominal pain as a neonate. There was no neonatal hypotonia and he had normal developmental milestones. At 20 months of age, mild hepatomegaly of 5-6 cm with increased echogenicity of the liver but no splenomegaly or kidney involvement was incidentally found on ultrasound. Laboratory results included elevated triglycerides at 3 mmol/L, alanine aminotransferase (ALT; NR 7–40) at 216 U/L and creatine kinase (CK; NR 40–320) at 1077 U/L. Liver biopsy showed mild portal fibrosis, and glycogen and lipid accumulation in some hepatocytes. A provisional diagnosis of glycogen storage disease (GSD) type IX was made despite the absence of hypoglycaemia, hyperlacticidaemia and hyperuricidaemia. Glucose-6-phosphate activity was low in the liver, which was in keeping with GSD type IX, however no pathogenic variants were identified in *PHKB* or *PHKG2*. At this time, no concerns were raised about his mental and physical development. No amendments to his diet were introduced.

His hepatomegaly resolved by 12 years of age, but CK remained raised concomitantly with ALT, indicating a developing muscle disorder. Differential diagnosis included other inherited metabolic diseases such as Pompe disease. Alpha-glucosidase activity in lymphocytes was however reported as normal.

Throughout childhood, he had frequent atopic allergic rhinitis treated with antihistamines. When reviewed at age 10, his longstanding ichthyosiform erythroderma was accentuated and widespread and refractory to emollients and topical hydrocortisone and emollient cream.

During this period, he was on combined steroids and long-acting beta-2 agonist inhalers for asthma but required additional salbutamol most days.

He had intermittent abdominal pain with occasional vomiting and had an irregular bowel habit throughout his childhood.

By the age of 14, his exercise tolerance was reduced, compounded by uncontrolled asthma, and profound distal muscle weakness. His neurological examination was otherwise unremarkable. The patient reported occasional post-exertional myalgia and increased fatigability. He subsequently reported progressive generalized weakness and started avoiding exercise.

His persistently low neutrophil count led to further haematological investigations. Blood film analysis revealed marked vacuolation of neutrophils, consistent with Jordan's anomaly. The association with the clinical history of ichthyosis, raised CK and new onset myopathy led to the clinical diagnosis of NLSD. Lipid vacuoles were also seen in eosinophils and monocytes in the same peripheral blood smear.

The patient and his parents underwent genetic analysis of the whole *PNPLA2* and *ABHD5* coding regions. Sequencing identified homozygous c.700C > T pathogenic variant in *ABHD5* in her son, while *PNPLA2* contained only known polymorphisms and no known pathogenic variants. *ABHD5* c.700C > T causes a premature stop codon at amino acid Arg234 and was found in heterozygous state also in both parents. This pathogenic variant has been previously detailed by Pike *et al* (2011).

Following the diagnosis, the patient was started on a diet free of long-chain fats which did not improve his symptoms.

Similar vacuoles were present in various skin biopsies, including one from his eyelid and another obtained later from an area of eczema on his right upper arm, explaining the treatment resistant ichthyosis. Additional biopsies from skeletal muscle (biceps femoris), gastrointestinal and respiratory mucosa were obtained to correlate symptoms with the underlying disorder. Absence of cardiac symptoms supported the ichthyotic NLSD diagnosis, as opposed to cardiomyopathic NLSD.

**Fig. 1:**
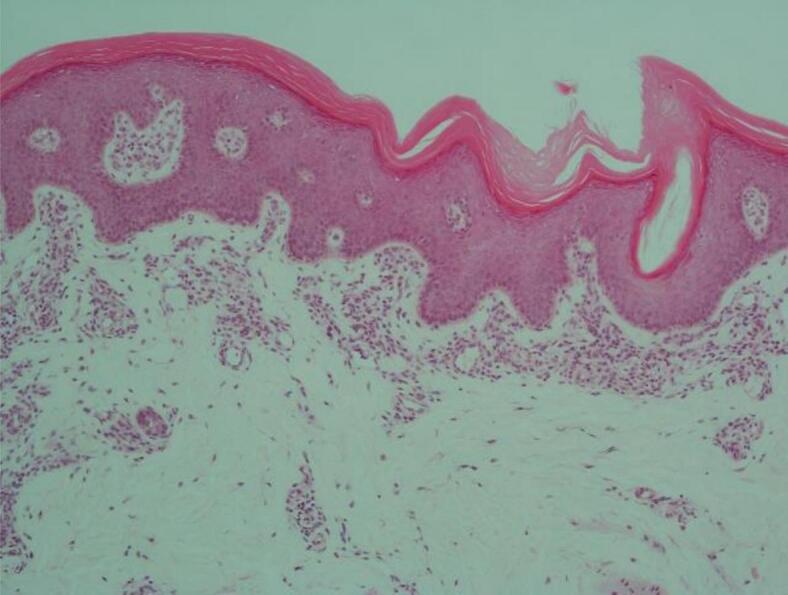
Arm skin biopsy showing epidermal acanthosis and slight papillomatosis together with overlying compact hyper- and parakeratosis and a preserved granular layer compatible with the variant of ichthyosis seen in NLSD (haematoxylin and eosin stain, original magnification x 100).

At age 16 years, the patient's care was transferred to the Adult Metabolic Clinic. Whilst gastrointestinal symptoms had improved, ichthyosis had worsened. Skin biopsy of his right arm showed features of a non-bullous congenital ichthyosiform erythema type as described in NLSD patients (Fig. 1). Acitretin was trailed by dermatology after much consideration, in view of his raised ALT level, however this was discontinued due to acute deterioration of liver function tests.

Since the diagnosis of CDS, he was advised to follow a low-fat diet (<10 g fat/ day) supplemented with medium chain triglycerides (MCT) oil. At the age of 18, this recommendation was adjusted to a fat intake of <20 g fat by the adult metabolic service to improve adherence to treatment. He continued to struggle to adhere to fat restriction and instead his diet was high in fat and sugar. He was also unable to tolerate MCT oil supplements and stopped taking walnut oil.

In his 20s, he reported subjective muscle weakness in his lower limbs and arms which stabilized with regular physiotherapy (Table 1). He could only walk for around seven minutes before having to stop because of fatigue and shortness of breath. At the last clinic review he walked independently, with no orthoses. Timed 10-m walk test was slightly slower than normal at 1.0 m/s (normative values for males in their 20s: 1.358 m/s). On examination, he had mild proximal weakness, but no muscle wasting or muscle hypertrophy.

**Table 1:** Muscle strength in patient over time.

Peak isometric contraction measured using myometry (lb)	RIGHT (Dominant) Aged 21	LEFT (Non-Dominant) Aged 21	RIGHT Aged 22	LEFT Aged 22	RIGHT Aged 25	LEFT Aged 25	RIGHT Aged 26	LEFT Aged 26
Shoulder Flexion	10.0	12.3	12.6	17.3	14.4	16.8	–	–
Elbow Flexion (Strength Ref Value Dominant- 64.0 lb. Non-Dom - 62.5 lb)	12.3	14.6	18.2	15.8		19.7	14.5	14.1
Hip Flexion (Strength Ref Value Dominant- 47.6 lb. Non-Dom- 46.5 lb)	16.8	15.9	19.4	16.1	16.7	13.2	8.0	6.8
Hip Abduction (Strength Ref Value Dominant- 72.2 lb. Non-Dom- 70.1 lb)	14.1	15.3	13.1	11.0	16.8	19.6	11.7	13.4
Hip Adduction	18.2	18.1	11.1	11.3	13.3	10	13.8	11.8
Knee Flexion	17.0	19.2	14.7	18.4	17.5	18.8	8.3	7.1
Knee Extension (Strength Ref Value Dominant- 129.3 lb. Non-Dom- 130.1 lb)	27.9	22.9	16.8	14.2	30.9	37	16.0	12.2

**Fig. 2:**
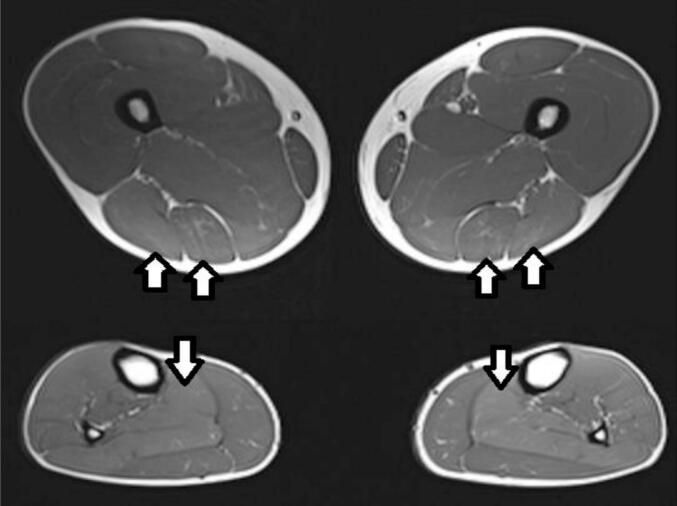
Axial T1 weighted MR images of the thighs (top) and calves (bottom). Subtle homogenous increased T1 signal within the biceps femoris, semimembranosus and soleus muscles bilaterally (arrows).

**Fig. 3:**
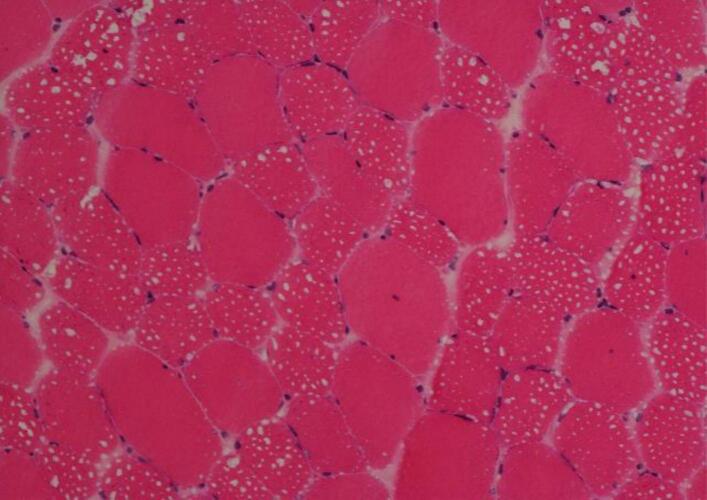

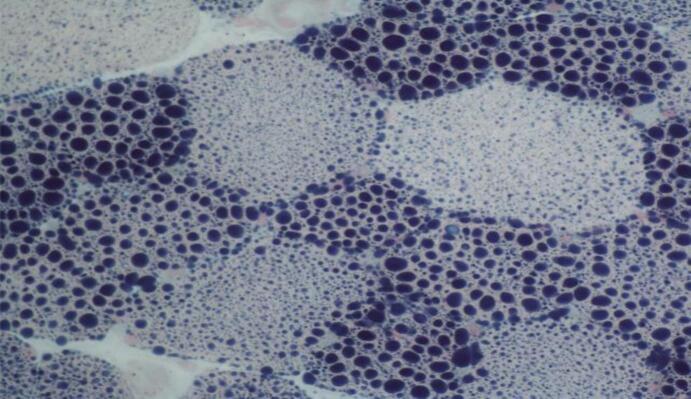

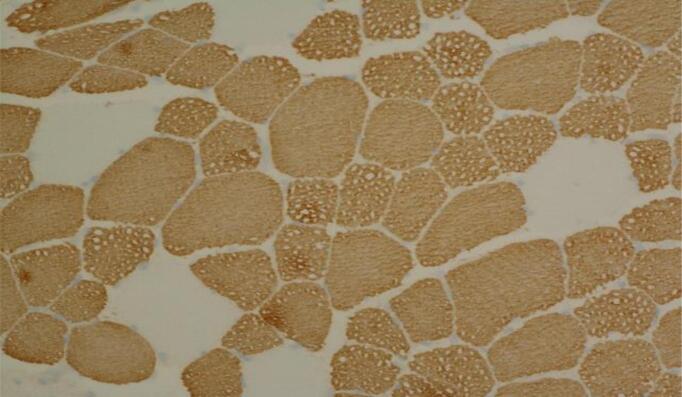
a.Thigh (left biceps femoris) muscle biopsy showing coarse vacuolation of a subset of muscle fibres (Haematoxylin and eosin stain, original magnification x 200).Fig. 3b Muscle biopsy showing numerous coarse lipid containing vacuoles in a population of smaller type 1 fibres; lipid accumulation in larger type 1 fibres is less obvious (Sudan black stain, original magnification x 400). Fig. 3c Thigh muscle biopsy showing a majority of type 1 fibres with more obvious vacuolation of smaller type 1 fibres (myosin heavy chain slow fibre immunostaining, original magnification x 200).

He reported significant myalgia and was prescribed various chronic pain medications (MCT oil, vitamin E, ursodeoxycholic acid, riboflavin (vitamin B2), and co-enzyme Q10). He tried bezafibrate 400 mg for 6 months intermittently with no improvement in his muscle symptoms, although there was objective improvement in his muscle strength after vitamin E and ursodeoxycholic acid administration (see Table 1). He tried gabapentin and pregabalin with no significant improvement in his symptoms. He continued vitamin D 2000 international units daily. A thigh and calf muscle MRI was performed (Fig. 2.) as well as a biceps femoris muscle biopsy (Fig. 3). EMG examination of selected muscles in the right lower and upper limbs and lumbar paraspinal muscles did not show any evidence of a significant underlying myopathy.

The muscle biopsy showed abnormal fibre size variation, several fibres with internal nuclei, hypotrophic and atrophic fibres and prominent sarcoplasmic lipid accumulation (Fig. 3) with more obvious vacuoles seen in hypotrophic or atrophic type 1 fibres (Fig. 3b and 3c). Sudan black demonstrated abnormally increased lipid store in both type 1 and type 2 fibres.

**Fig. 4:**
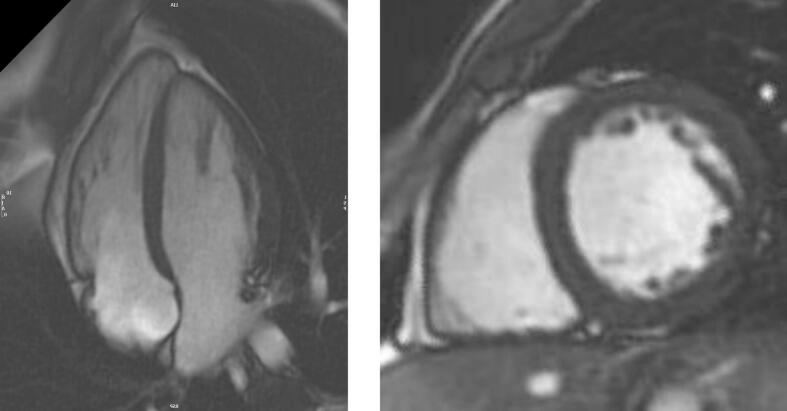

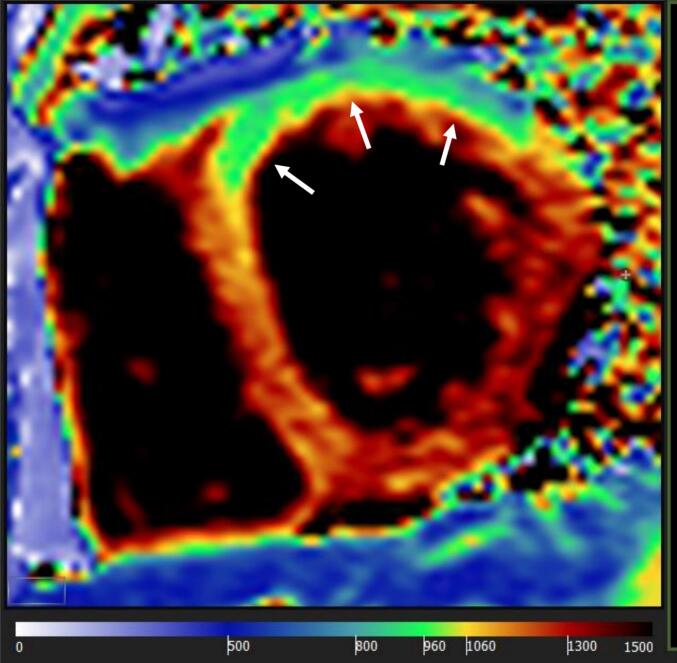

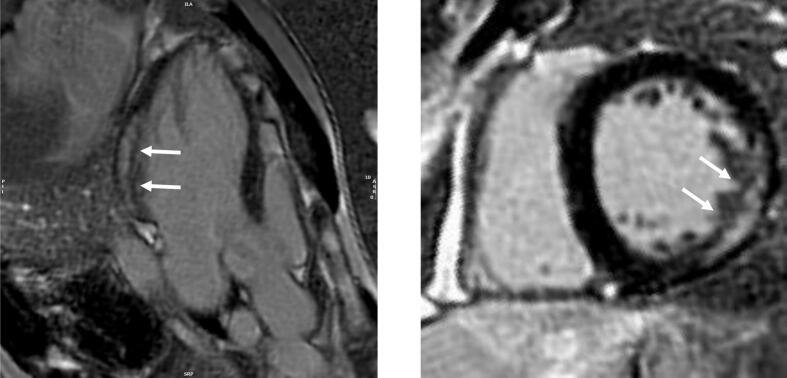
Cardiac MRI Fig. 4a-b. Steady state free precession cine imaging (4-chamber view; left panel and mid short axis, right panel), showing left ventricular myocardial thickness within normal limits (interventricular septum thickness = 10 mm, lateral wall thickness = 7 mm).c Native T1 mapping (mid short-axis slice) showing high native T1 values (ranging from 1100 ms to 1350 ms, suggesting high interstitial component secondary to fibrosis) with the exception of the anterior/anterolateral segments (white arrows), where native T1 values are low (920–930 ms, suggesting intramyocardial lipid accumulation).d-e. Phase sensitive inversion recovery late gadolinium enhancement imaging (4-chamber view, left panel, and mid short-axis view, right panel) showing dense intramyocardial enhancement in the basal-mid inferolateral segments (white arrows), indicating replacement fibrosis.

At the age of 23, his liver function tests showed deranged ALT between 73 and 173 U/L, which was in keeping with his persistently raised CK (700–3000 U/L), normal ALP and serum bilirubin. He tried ursodeoxycholic acid 300 mg daily and vitamin E 60 mg daily for almost a year, as well as Co-enzyme Q10 and riboflavin with no significant change in his liver function. His liver ultrasound confirmed fatty infiltration. Due to suffering with chronic fatigue this necessitated a break from his university course. There was no breathlessness to report and his pulmonary function test (FEV1 4.61 L, FEV1% predicted 127 %; FVC 5.74 L, FVC% predicted 134 %) and echocardiogram (IVSd 8 mm, LVIDd 52 mm, LVIDs 36 mm, LVPWd 11 mm and EF 57.9 %) were unremarkable. His cardiac MR however demonstrated changes in keeping with focal intramyocardial lipid accumulation and interstitial and replacement fibrosis (Fig. 4 a,b,c).

He started to engage with diet advice with the aim of optimising symptom management of his metabolic condition. He now uses MCT oil in cooking and has reduced his intake of high fat, high sugar foods and drinks. He has a regular intake based around 3 to 4 meals a day). He was also taking co-enzyme Q10 and riboflavin at this time. He aims for a low-fat intake (< 30 g fat daily) and has an estimated 1.4 g/kg/day protein. He has observed stabilisation in some of his symptoms including improved concentration, wellbeing and energy levels. He has also reported the change in diet did lead to stabilisation of his muscle symptoms, although did not appear to improve his muscle strength long-term (See Table 1).

Strength Reference Values adapted from: Bohannon, R (1997) Reference values for extremity muscle strength obtained by hand-held dynamometry from adults aged 20 to 79 years. *Archives of Physical Medicine and Rehabilitation*. Vol 78 (1), 26–32 [40].

## 3 Discussion

### 3.1 Significance of the pathogenic variants

This case report confirms that patients with ichtyotic NLSD may develop myopathy later in life. The homozygous c.700C > T nonsense mutation in exon 5 of *ABHD5*/CGI-58 gene in our patient, has previously been reported in patients with progressive myopathy from Egypt [17] and China [18].

At the time, Sanger sequencing with multiple and/or bidirectional primers and analysis by two or more independent analysts was the gold standard for detecting pathogenic variants and remains a high-quality method for targeted analysis when the diagnosis is suspected.

### 3.2 Myopathy

The CGI-58 protein binds cytoplasmic lipid molecules to initiate the hydrolysis of triacylglycerols [1]. How CGI-58 further promotes metabolism of stored fats is not yet well-elucidated [19].

CGI-58 upregulates perilipin and adipose triglyceride lipase activity, transporting released fatty acids. It is a member of the large α/β-hydrolase family of proteins with 349 amino acids and a molecular mass of approximately 39 kD that enables the activation of ATGL which plays and important role in intracellular lipolysis. Schweiger et al. [6] reported that the α/β hydrolase domain contains the putative activation region of ATGL^.^ Pathogenic variants in the *ABHD5* gene results in the total or partial inactivation of the ATGL enzyme that enables the release of fatty acids (FAs) from the intracellular triacylglycerol (TG) stores of adipose and non-adipose tissues. This leads to insufficient FA mobilisation within the cell, which in turn causes the accumulation of intracytoplasmic lipid droplets that contain TG [1,20].

Patients with defective ATGL demonstrate more severe myopathy and patients with pathogenic variants in ABHD5/CGI-58, which has the ATGL-activating function, always present with ichthyosis [6]. The mechanisms of ichthyosis in NLSD is postulated to be impaired acylceramide production, essential for skin permeability barrier formation, as observed in ABHD5 knockout mice [21]. The patatin-like phospholipase domain containing protein 1 (PNPLA1) catalyzes the final step of acylceramide synthesis [21]. Clinical symptoms of NLSD however cannot be explained by cooperation between ABHD5 and PNPLA1 or ATGL/PNPLA2 alone. Therefore, it is possible that ABHD5 interacts with other PNPLA family proteins [21].

In muscle cells, cytoplasmic FA containing lipid droplets are an easily accessible source of energy. The decrease in the efficiency of ATGL lipolysis due to *ABDH5* mutation leads to two different forms of NLSD, which are the early onset form that is accompanied by ichthyosis, and the adult-onset progressive myopathy. The decrease in efficiency of ATGL lipolysis due to the ABDH5 mutation results in lipid droplet accumulation in both type 1 and type 2 muscle fibres. Lipid droplets are commonly used in phospholipid synthesis, so their deficiency leads to phospholipid membrane abnormalities. This disorder results in myopathy in up to 59 % of cases [1,22].

CDS myopathy tends to manifest as a slowly progressive muscle weakness; cardiomyopathy is rare. Myopathic NLSD patients often show progressive muscular atrophy, which is not described in CDS patients [2]. Additionally, the lipid accumulation is probably lower in the epidermis of myopathic NLSD patients.

### 3.3 Cardiac fibrosis

In a recent study of 9 Iranian NLSD patients, 2 died of cardiomyopathy aged 29 and 43 respectively, with onset of weakness being only a couple of year before [23]. Cardiomyopathy often presents in the fourth to sixth decade of life, however cardiac MS spectroscopy has shown that intracellular lipid levels are up to 10 times age matched controls, even in the second decade of life [24] and there are reports of dilated cardiomyopathy, as young as age 19 [25]. In the Iranian cohort, both patients were asymptomatic from a cardiac point of view or may present with vague symptoms like limb weakness [26], and in fact one patient had a normal echocardiogram 1 year before their death [23] therefore, cardiac MRI is strongly recommended, both at diagnosis and periodically afterwards to detect early myocardial lipid deposition and prevent fibrosis.

### 3.4 Role of skin biopsy

This patient's blood film previously confirmed the presence of Jordan’ anomaly [8]. Vacuolated leucocytes are present in several conditions such as multiple sulfatase deficiency, ichthyosis sclerosing cholangitis syndrome, systemic carnitine deficiency, Refsum disease and Wolman disease. However, the presence of congenital ichthyosis with systemic neutral lipid deposition is typical of CDS. It is a typical feature of CHKB related muscular dystrophy, another rare neuromuscular disorder [27].

Although the ichthyosiform phenotype in ichtyotic NLSD is nonspecific, most closely resembling non-bullous congenital ichthyosiform erythroderma, patients often experience pruritus, with or without atopic features [28]. Skin biopsy is not essential but may provide additional information during diagnostic work-up and often demonstrates lipid droplets *in vitro*, most prominently in the epidermal basal layer and in appendageal epithelia [29]. However, such storage vacuoles are not likely to account for the ichthyosiform phenotype in NLSD, because cytosolic inclusions become covered within corneocytes, where they cannot influence inherently extracellular functions, such as permeability barrier homeostasis or desquamation. Moreover, comparable cytosolic lipid droplets occur as a nonspecific response to toxic insults, as well as in many hyperplastic dermatoses [30,31] The severity of the skin condition is not correlated with the presence of lipid droplets in the epidermis [32].

### 3.5 Brain magnetic resonance spectroscopy as a biomarker

Lipid peaks detected at brain 1H-MR spectroscopy can be an additional tool of CDS [33]. Clinical evidence is insufficient to determine if lipid accumulation in the brain can be considered a sensitive biomarker [33]. It has been hypothesized that the occurrence of cerebral lipid accumulation in CDS directly relates to the same underlying pathophysiologic mechanism and that the MRS lipid peak in striking lipid accumulation in the cerebral white matter, cortex and basal ganglia derives from triacylglycerol [33].

### 3.6 Liver involvement

Diagnosis of CDS is sometimes delayed, with the diagnosis being established late in the course of the disease. Late diagnosis is associated with significantly high morbidity and mortality [12], mainly caused by advanced liver disease. Most patients have liver involvement with evidence of cirrhosis in advanced age. Frequent monitoring with liver USS and MRI scans as well as fibroscan will help identify subtle changes in steatohepatitis and the progression to liver cirrhosis, which has been documented in up to 11–15 % [1,3].

### 3.7 Treatment

No specific pharmacologic treatment is available for CDS. A low-fat diet supplemented with MCT, ursodeoxycholic acid and vitamin E were recommended for patients with liver involvement. In this case there were initial improvements in muscle strength and in energy levels, well-being and muscle symptoms with these treatments. Retinoids such as acitretin, which are the systemic therapeutic modality of choice in lamellar ichthyosis, are found to be useful in the treatment of skin and muscle manifestations in CDS [34,35], however they cannot be given in the presence of impaired liver function, which is common in CDS. Triheptanoin, a synthetic medium-chain triglyceride, has shown a beneficial effect on muscle function in myopathic NSLD [34,35]. Triheptanoin produces acetyl-CoA and propionyl-CoA, the later of which can be further converted into succinyl-CoA, an anaplerotic substrate for the tricarboxylic acid cycle, supporting mitochondrial energy production.

Reduction of hepatomegaly and normalization of liver enzymes has been observed with dietary modifications [36,37]. Additional studies are required to clarify the benefits of low-fat diet in CDS patients. In our patient, myopathy did not clearly respond to MCT product.

More recently liver transplant has been used to treat the liver complications of CDS successfully [38] and *in vivo* studies with CDS skin fibroblasts have suggested that malaria drug, tafenoquine, the anti-oxidant, MitoQ and lipid lowering therapy, Lomitapide are effective in reducing lipid droplet area and total numbers and could be potential future treatments [39]. Interestingly in this case there was a stabilisation in energy, well-being and muscle symptoms whilst he was taking co-enzyme Q10, another form of MitoQ, although he was also engaging with diet and use of MCT oil during this time, so improvements could also be attributed to this.

## 4 Conclusion

In conclusion, the molecular mechanisms underlying the functions of the CGI-58 protein are complex. In addition, CGI-58 seems to have multiple targets, which are a likely explanation of the multiple clinical manifestations of CDS. Further understanding of the mechanism of CGI-58-driven triglycerides hydrolysis in each cell type and ATGL-independent actions, would explain the molecular basis of and the optimal approach for the clinical treatment of CDS.

Cardiac MR exam was performed on 1.5 T Avanto scanner, Siemens, Munich, Germany) and included steady-state free precession cine imaging (standard long- and short-axis views), basal and mid LV short-axis native T1 mapping (Modified Look-Locker Inversion Recovery) and phase sensitive inversion recovery late gadolinium enhancement (LGE) imaging acquired 15 min post-administration of gadolinium-based contrast agent (0.1 mmoL/kg gadobutrol [Gadovist, Bayer AG]).

## Details of funding

N/A.

## Details of ethics approval

N/A.

## A patient consent statement

Patient's consent was obtained.

## Documentation of approval from the Institutional Committee for Care and Use of Laboratory Animals (or comparable committee)

N/A.

## CRediT authorship contribution statement

**Kinza Noman:** Writing – review & editing, Writing – original draft, Methodology, Formal analysis, Data curation. **Andreas Tridimas:** Writing – review & editing, Writing – original draft, Methodology, Formal analysis, Data curation. **James B. Lilleker:** Writing – review & editing, Writing – original draft, Methodology, Formal analysis, Data curation. **Gaetano Nucifora:** Writing – review & editing, Writing – original draft, Methodology, Formal analysis, Data curation. **Peter Woolfson:** Writing – review & editing, Writing – original draft, Methodology, Formal analysis, Data curation. **Daniel du Plessis:** Writing – review & editing, Writing – original draft, Methodology, Formal analysis, Data curation. **Alison Woodall:** Writing – review & editing, Writing – original draft, Methodology, Formal analysis, Data curation. **Andrew Oldham:** Writing – review & editing, Writing – original draft, Methodology, Formal analysis, Data curation. **Mark E. Roberts:** Writing – review & editing, Validation, Methodology. **John Bassett:** Writing – review & editing, Project administration, Methodology, Data curation. **Federico Roncaroli:** Writing – review & editing, Writing – original draft, Methodology, Formal analysis, Data curation. **Simon A. Jones:** Writing – review & editing, Validation, Methodology. **Stefan Coassin:** Validation, Methodology. **Florian Kronenberg:** Validation, Methodology. **Karolina M. Stepien:** Writing – review & editing, Validation, Resources, Conceptualization.

## Declaration of competing interest

A competing interest statement: All authors declare no potential conflict of interest.

## Data Availability

No data was used for the research described in the article.
